# Low prevalence of hepatitis C co-infection in recently HIV-infected minority men who have sex with men in Los Angeles: a cross-sectional study

**DOI:** 10.1186/s12879-015-1279-z

**Published:** 2015-11-20

**Authors:** Kara W. Chew, Martha L. Blum, Marjan Javanbakht, Laurel E. Clare, Lorelei D. Bornfleth, Robert Bolan, Debika Bhattacharya, Pamina M. Gorbach

**Affiliations:** Department of Medicine, David Geffen School of Medicine at UCLA, 11075 Santa Monica Blvd, Suite 100, Los Angeles, CA 90025 USA; Department of Medicine, David Geffen School of Medicine at UCLA, 10833 Le Conte Ave, 37-121 CHS, Los Angeles, CA 90095 USA; Present Address: Community Hospital of the Monterey Peninsula, Infectious Diseases Division, 23625 Holman Highway, Monterey, CA 93942 USA; Department of Epidemiology, UCLA Fielding School of Public Health, 71-235E CHS, Los Angeles, CA 90095 USA; Los Angeles LGBT Center, 1625 N. Schrader Blvd, Los Angeles, CA 90028 USA; Department of Medicine, David Geffen School of Medicine at UCLA, 41-295 CHS, Los Angeles, CA 90095 USA

**Keywords:** Hepatitis C, HIV, Transmission, Non-injection drug use, Men who have sex with men

## Abstract

**Background:**

Geographic and sociodemographic characterization of hepatitis C virus (HCV) transmission amongst men who have sex with men (MSM) has been limited. Our aim was to characterize HCV prevalence, risk factors for HCV co-infection, and patterns of HIV and HCV co-transmission and transmitted drug resistance mutations (DRMs) in newly HIV-diagnosed Los Angeles MSM.

**Methods:**

Viral RNA was extracted from stored plasma samples from a Los Angeles cohort of newly diagnosed HIV-infected MSM with well-characterized substance use and sexual behavioral characteristics via computer-assisted self-interviewing surveys. Samples were screened for HCV by qPCR. HCV E1, E2, core, NS3 protease and NS5B polymerase and HIV-1 protease and reverse transcriptase regions were amplified and sequenced. Phylogenetic analysis was used to determine relatedness of HCV and HIV-1 isolates within the cohort and viral sequences were examined for DRMs.

**Results:**

Of 185 newly HIV-diagnosed MSM, the majority (65 %) were of minority race/ethnicity and recently infected (57.8 %), with median age of 28.3 years. A minority (6.6 %) reported injection drug use (IDU), whereas 96 (52.8 %) reported recent substance use, primarily cannabis or stimulant use. High risk sexual behaviors included 132 (74.6 %) with unprotected receptive anal intercourse, 60 (33.3 %) with group sex, and 10 (5.7 %) with fisting. Forty-five (24.3 %) had acute gonorrhea or chlamydia infection. Only 3 (1.6 %) subjects had detectable HCV RNA. Amongst these subjects, HIV and HCV isolates were unrelated by phylogenetic analysis and none possessed clinically relevant NS3 or NS5B HCV DRMs.

**Conclusions:**

Prevalence of HCV co-infection was low and there was no evidence of HIV-HCV co-transmission in this cohort of relatively young, predominantly minority, newly HIV-diagnosed MSM, most with early HIV infection, with high rates of high risk sexual behaviors, STI, and non-IDU. The low HCV prevalence in a group with high-risk behaviors for non-IDU HCV acquisition suggests an opportune time for targeted HCV prevention measures.

## Background

Hepatitis C virus (HCV) co-infection is common amongst HIV-infected persons, affecting an estimated 4 to 5 million persons worldwide, and is associated with increased morbidity and mortality [1, 2]. Whereas the primary route of HCV transmission remains injection drug use (IDU), over recent years there has been increasing evidence of sexual transmission among HIV-infected men who have sex with men (MSM), likely driven by mucosal risk factors, including unprotected and traumatic sexual practices in the context of multiple partners, non-injection drug use, and sexually transmitted infections [3–7]. Prevalence estimates for HCV co-infection in HIV-infected MSM have ranged from 6 to 15.7 %, with limited geographic characterization [8–12]. The prevalence of HCV co-infection in HIV-infected MSM in Los Angeles County (LAC) in the U.S. has not been defined, despite LAC being the second largest epicenter for AIDS cases nationally, with high rates of non-injection drug use and high-risk sexual practices. Our aims were to characterize the prevalence of and risk factors for HCV co-infection and patterns of HIV and HCV co-transmission and drug resistance mutations (DRMs) in a cohort of newly HIV-infected or HIV-diagnosed Los Angeles MSM.

## Methods

Between February 2009 and May 2012 we enrolled participants from a community-based organization providing sexual health services to the gay community in LAC: The Los Angeles LGBT Center. Clients were eligible if they were recently HIV-infected (<1 year of infection) or newly HIV-diagnosed, at least 18 years of age, male, and reported sex with a male partner in the past year. Recent infection was determined by one of four ways: nucleic acid amplification test with negative serology, detuned assay (Low Sensitivity Vitros ECi, Ortho Clinical Diagnostics, Rochester, NY), provider verification, or negative HIV testing within the past year. Plasma was collected and syphilis, chlamydia, and gonorrhea testing performed for all participants. Behavioral and sociodemographic data were collected via computer-assisted self-interview. Minority race/ethnicity was defined as self-identified African-American race or Hispanic ethnicity.

Viral RNA was extracted from stored plasma using the UltrasSens Viral Isolation kit (Qiagen), converted to cDNA using Superscript III Reverse Transcriptase (Invitrogen), and screened for HCV by quantitative polymerase chain reaction (PCR) designed to detect the HCV 5′UTR. The HCV E1/E2/core, NS3 and NS5B regions from HCV positive samples and HIV-1 protease and RT regions from all samples were amplified using either Phusion High-Fidelity DNA Polymerase (New England BioLabs) or KOD High-Fidelity Polymerase (Novagen). PCR products were directly sequenced using the BigDye v3.1 Kit (Applied Biosystems).

Sequences were checked for quality, edited, and alignments performed using Chromas Lite (Technelysium Pty Ltd, South Brisbane, Australia) and BioEdit (Ibis Biosciences, Carlsbad, CA). Consensus sequences of an 857 nucleotide (nt) fragment of the HCV NS3 protease gene spanning amino acids 13–297 were aligned with genotype consensus sequences obtained from the Los Alamos National Laboratory (LANL) HCV database (http://www.hiv.lanl.gov), JFH-1, and additional HCV sequences collected from the Los Angeles area. A 1302 nt fragment of the *pol* gene (HXB2 reference location nt 2254–3555) was aligned with the Clade B consensus sequences obtained from the LANL HIV database. Neighbor-joining phylogenetic trees were generated using the DNAdist and Neighbor programs of PHYLIP. Sequences were examined for the following HCV DRMs: V36M/A, T54A/S, V55A, Q80K, R155K, A156S/T, D168T/V, I/V170A and S282T. HIV-1 DRMs were determined using the Stanford University HIV Drug Resistance Database (http://hivdb.stanford.edu/).

The study was reviewed and approved by the University of California, Los Angeles Institutional Review Board (IRB# 10–000806) and the Los Angeles LGBT Center Review Committee. Written informed consent was obtained from each study participant, including permission to use stored plasma samples for research testing.

## Results

One hundred eight-five subjects were included in this study. Sociodemographic, behavioral, and clinical characteristics are summarized in Table 1. Median age (interquartile range, IQR) was 28.3 (24.7–35.0 years) and the majority were of minority race or ethnicity (66.9 %) and recently HIV-infected (57.8 %). At the time of plasma collection, 24 (13.7 %) reported being prescribed antiretroviral therapy (ART). Median number of partners within the past 12 months was 9 (IQR 4–20), with unprotected receptive or insertive anal intercourse (URAI or UIAI) reported by 132 (74.6 %) and 115 (64.6 %), respectively. Forty-five (24.3 %) subjects tested positive for gonorrhea or chlamydia, with 47 (27.5 %) testing positive for syphilis at baseline. A minority (6.6 %) reported IDU in the past 12 months, whereas 96 (52.8 %) reported recent substance use, primarily cannabis or stimulant use.

**Table 1:** Baseline characteristics of the cohort, overall and by hepatitis C virus (HCV) status

Characteristic	Total (*N* = 185) n^a^ (%) or median (IQR)	HCV negative (*N* = 182) n^a^ (%) or median (IQR)	HCV positive (*N* = 3) n^a^ (%) or median (IQR)
Sociodemographics			
Age (years)	28.3 (24.7–35.0)	28.3 (24.7–34.8)	35.8 (22.3–45.1)
Race			
African American	32 (17.7)	31 (17.4)	1 (33.3)
Hispanic	89 (49.2)	87 (48.9)	2 (66.7)
White	47 (26.0)	47 (26.4)	0 (0)
Asian	5 (2.8)	5 (2.8)	0 (0)
Other	8 (4.4)	8 (4.4)	0 (0)
Incarcerated, past 12 months	17 (9.3)	17 (9.5)	0 (0)
Substance Use, past 3 months			
Any drugs (including cannabis)	96 (52.8)	93 (52.0)	3 (100)
Cannabis	79 (43.4)	77 (43.0)	2 (66.7)
Cocaine	26 (14.3)	25 (14.0)	1 (33.3)
Methamphetamine	33 (18.1)	30 (16.8)	3 (100)
Inhalants	6 (3.3)	5 (2.8)	1 (33.3)
Sedatives	16 (8.8)	15 (8.4)	1 (33.3)
Hallucinogens	3 (1.7)	2 (1.1)	1 (33.3)
Opioids	6 (3.3)	5 (2.8)	1 (33.3)
Injection drug use, past 12 months	12 (6.6)	11 (6.2)	1 (33.3)
Partner injection drug use, past 12 months	34 (18.6)	32 (17.8)	2 (66.7)
Alcohol use, past 3 months	129 (70.9)	127 (71.0)	2 (66.7)
Sexual Behaviors			
Sexual partners, past 12 months	9 (4–20)	9 (4–20)	6 (6–14)
Unprotected insertive AI, last 6 partners	115 (64.6)	113 (64.2)	2 (66.7)
Unprotected receptive AI, last 6 partners	132 (74.6)	130 (74.3)	2 (66.7)
Fisted by partner, last 6 partners	10 (5.7)	10 (5.8)	0 (0)
Transactional sex	20 (11.0)	19 (10.6)	1 (33.3)
Group sex	60 (33.3)	58 (32.4)	2 (66.7)
Sexually Transmitted Infections			
Chlamydia, baseline testing	32 (18.2)	30 (17.3)	2 (66.7)
Gonorrhea, baseline testing	19 (10.6)	19 (10.8)	0 (0)
Syphilis, TPPA positive, baseline testing	47 (27.5)	46 (27.2)	1 (33.3)
Chlamydia, self-reported, past 12 months	69 (37.9)	68 (38.0)	1 (33.3)
Gonorrhea, self-reported, past 12 months	65 (35.7)	64 (35.8)	1 (33.3)
Syphilis, self-reported, past 12 months	57 (31.3)	56 (31.3)	1 (33.3)
HSV, self-reported, past 12 months	11 (6.0)	11 (6.2)	0 (0)
HIV viral load, copies/mL^b^	41,538 (5771–150,756)	41,518 (6296–144,378)	60,476 (<50–155,000)
CD4 cell count (cells/mm^3^)^c^	553 (408–692)	553 (408–692)	Unavailable

*IQR* interquartile range, *AI* anal intercourse, *TPPA* treponema pallidum particle agglutination, *HSV* herpes simplex virus

^a^Sum of n may not equal total N because of missing data; ^b^Available for 171 of 185 subjects; ^c^Available for 80 subjects

Only 3 (1.6 %) subjects had detectable HCV RNA. HCV viral load ranged from 67,000 to 2.2 million copies/ml. There were too few HCV infections to identify significant risk factors for HCV co-infection. Of the 3 HCV-positive subjects, all were of minority race or ethnicity; one subject was classified as newly HIV infected by detuned assay and two were newly diagnosed with HIV infection of unknown duration. Only 1 reported a history of IDU. All 3 subjects reported non-injection drug use, including methamphetamines, within the past 3 months. All 3 subjects also reported high-risk sexual behavior, including UIAI, URAI, and group sex.

**Fig. 1:**
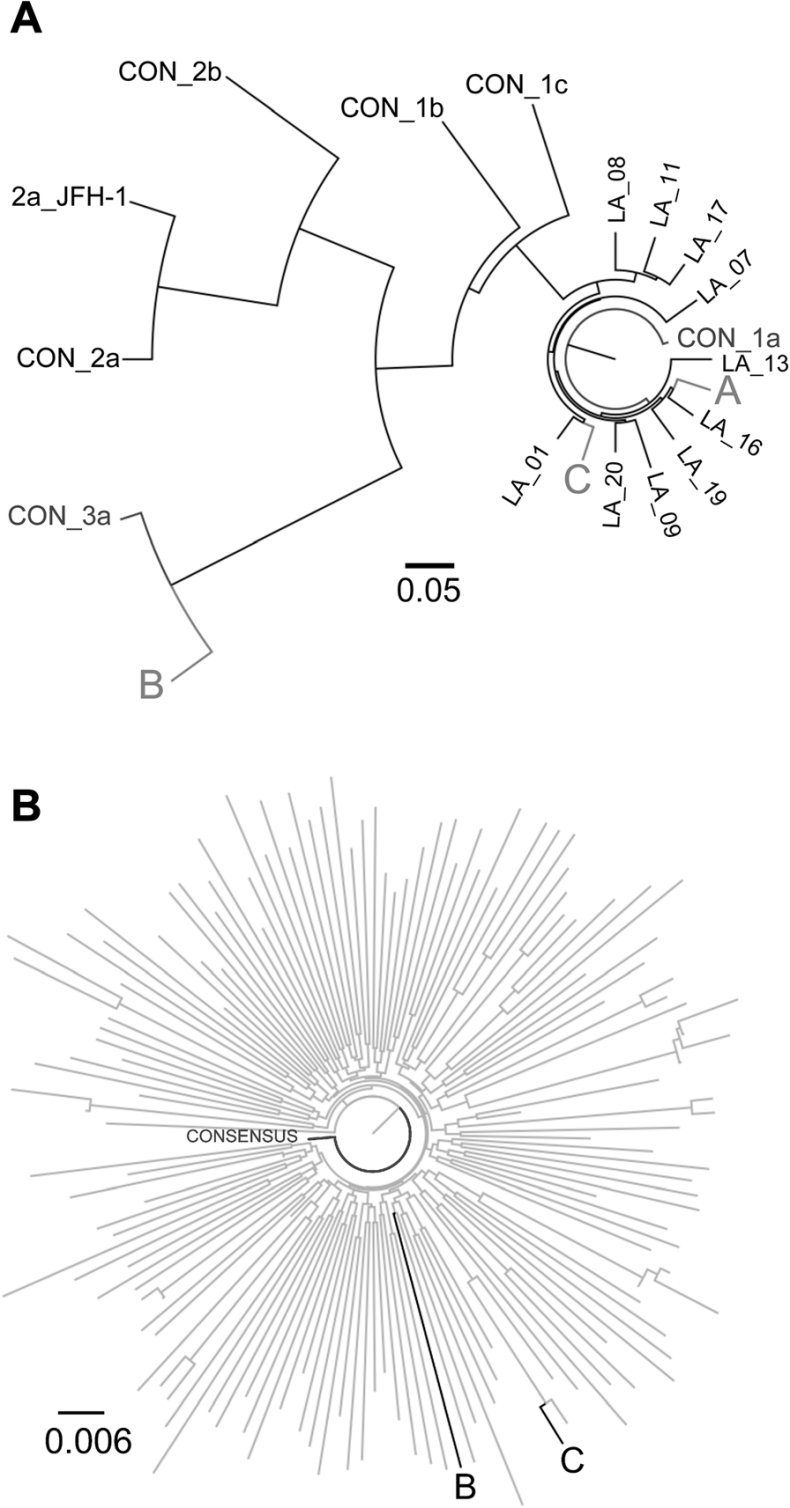
**a** Phylogenetic analysis of Hepatitis C virus (HCV) isolates. Consensus sequences of an 857 nucleotide (nt) fragment of the HCV NS3 protease from the three HCV isolates from the cohort were aligned with the Los Alamos National Laboratory HCV Database consensus sequences for HCV genotype (gt) 1a and 3a, along with additional reference sequences from subjects living in Los Angeles, to make a neighbor-joining tree. The consensus sequences for gt 1a and 3a are labeled as “CON” with the corresponding genotype. The Los Angeles HCV sequences are labeled as “LA” with the corresponding subject number. The HCV-positive subject samples are labeled A, B, and C. The tree is rooted with the HCV genotype 1a consensus sequence and the genetic distance scale bar is located at the center of the figure. **b** Phylogenetic analysis of HIV-1 *pol*. A 1302 nt fragment of *pol* covering the HIV-1 protease and reverse transcriptase (HXB2 reference location nt 2254–3555) from *N* = 148 isolates from the cohort was aligned with the Clade B consensus and used to build a neighbor-joining tree. Each sequence is labeled with a unique subject identifier, and the HCV-positive subjects are labeled A, B, and C. The tree is rooted with the Clade B consensus sequence. The genetic distance scale bar is located at the lower left of the figure

Amongst the 3 HCV-infected subjects (subjects A, B, and C), HIV and HCV sequences were unrelated by phylogenetic analysis (see Fig. 1). Based on HCV NS3 protease sequences, a neighbor-joining phylogenetic tree showed that HCV sequences from subjects A and C were most closely related to genotype 1a; and subject B, to genotype 3a. The two genotype 1a-infected subjects had sequences that were no more closely related to each other than to other isolates from the Los Angeles area, indicating that these were not a closely linked transmission pair. Similar results were obtained using a 1784 nt fragment of HCV NS5B and a 2470 nt fragment spanning the E1-E2-Core region of the genome (data not shown). No HIV-1 sequence was obtained from HCV-positive subject A, who was identified at screening as having recently acquired HIV infection, but had initiated antiretroviral therapy with fully suppressed HIV viremia at the time of study enrollment and plasma collection. HIV-1 sequences from subjects B and C were no more closely related to each other than to sequences obtained from other cohort members. However, the HIV-1 sequences from subject C and another subject (D) were closely related with 100 % bootstrap support and an inter-patient genetic distance of <1 % strongly indicative of these being a transmission pair. Subject D was HCV-negative, both by qPCR and serologically. One subject, who was ART naïve, had an HIV RT mutation (K103N). None had clinically relevant NS3 or NS5B HCV DRMs.

## Discussion

Prevalence of HCV co-infection was low and there was no evidence of HIV-HCV co-transmission in this cohort of young, predominantly minority, newly HIV-diagnosed MSM. The majority of subjects had recent HIV infection and notable behavioral and clinical risk factors for sexual HCV transmission, including high-risk sexual practices, sexually transmitted infections, and non-injection (primarily stimulant) substance use, with low rates of injection drug use. The lower prevalence of HCV compared with other HIV-infected MSM cohorts (1.6 vs 6–15.7 %) [8–12] may reflect the younger age of the cohort with fewer cumulative exposures to HCV, lower rates of IDU, relatively greater immune preservation with earlier HIV infection, and identification of HCV by HCV RNA instead of by serology. In our study, by measuring HCV RNA, we measured prevalence of active HCV replication and not exposure or infection with possible clearance, as would be measured by serology. Assessment by both serology and HCV RNA would provide broader characterization of HCV exposure in the cohort, but due to limited sample volume, we could not perform testing for both and elected for HCV RNA testing alone as a measure of active HCV infection and risk for HCV transmission. Demographically, our cohort differed from others in its geographic and racial/ethnic composition, wherein our cohort was predominantly of minority race and half was Hispanic, as compared to most other reported cohorts that were predominantly White. The epidemiology of HCV co-infection in HIV-infected Hispanic MSM has not been well described. As described by Kunikholm et al., utilizing National Health and Nutrition Examination Survey (NHANES) and Hispanic Community Health Study/Study of Latinos (HCHS/SOL) data, HCV prevalence appears to differ by Hispanic/Latino background and the prevalence of HCV in the West Coast Hispanic population may be lower than in others [13].

While there were too few subjects with HCV infection in the cohort to explore associations between potential behavioral risk factors and HCV infection, the subjects that did have HCV co-infection all reported methamphetamine and other non-injection drug use, as well as high-risk sexual practices, consistent with risk factors identified in larger cohorts. The lack of HCV clustering or transmission pairs is likely due to the smaller sample size than in other studies [6, 7], as well as may relate uniquely to the geographically wide sexual networks in LAC compared to other urban areas. The lack of evidence of HIV-HCV co-transmission may reflect the low prevalence of HCV infection in the cohort, but may also suggest that co-transmission occurs infrequently, and that HCV infection is more often acquired following HIV infection, instead of simultaneously or preceding HIV infection, particularly in non-IDU settings. The lack of HCV DRMs is likely due to the few observed HCV infections.

## Conclusions

We found a lower than expected prevalence of HCV infection in our LAC HIV-infected cohort of MSM. By behavioral risk factors, the population studied is at high risk for incident HCV infection. This presents an opportunity, namely at the time of a new HIV diagnosis and during early HIV infection, for targeted HCV prevention strategies to reduce non-IDU HCV transmission among HIV-infected MSM.

## Availability of data and materials

We are complying with the NIH policy on shared data. HIV and HCV sequences are available in the NIH genetic sequence database GenBank, accession numbers KJ680920 - KJ680970 and KM874782 - KM874789.
